# Machine Learning Model for Prediction of Development of Cancer Stem Cell Subpopulation in Tumurs Subjected to Polystyrene Nanoparticles

**DOI:** 10.3390/toxics12050354

**Published:** 2024-05-10

**Authors:** Amra Ramović Hamzagić, Marina Gazdić Janković, Danijela Cvetković, Dalibor Nikolić, Sandra Nikolić, Nevena Milivojević Dimitrijević, Nikolina Kastratović, Marko Živanović, Marina Miletić Kovačević, Biljana Ljujić

**Affiliations:** 1 Department of Genetics, Faculty of Medical Sciences, University of Kragujevac, Svetozara Markovića 69, 34000 Kragujevac, Serbia; ramovicamra@gmail.com (A.R.H.); marinagazdic87@gmail.com (M.G.J.); sandranikolic72@yahoo.com (S.N.); n_kastratovic@outlook.com (N.K.); bljujic74@gmail.com (B.L.); 2 Center for Harm Reduction of Biological and Chemical Hazards, Faculty of Medical Sciences, University of Kragujevac, 34000 Kragujevac, Serbia; 3 Institute for Information Technologies Kragujevac, University of Kragujevac, 34000 Kragujevac, Serbia; markovac85@kg.ac.rs (D.N.); nevena.milivojevic@uni.kg.ac.rs (N.M.D.); marko.zivanovic@uni.kg.ac.rs (M.Ž.); 4 Department of Histology and Embryology, Faculty of Medical Sciences, University of Kragujevac, 34000 Kragujevac, Serbia; marina84kv@gmail.com

**Keywords:** cancer stem cells, polystyrene nanoparticles, genetic algorithm

## Abstract

Cancer stem cells (CSCs) play a key role in tumor progression, as they are often responsible for drug resistance and metastasis. Environmental pollution with polystyrene has a negative impact on human health. We investigated the effect of polystyrene nanoparticles (PSNPs) on cancer cell stemness using flow cytometric analysis of CD24, CD44, ABCG2, ALDH1 and their combinations. This study uses simultaneous *in vitro* cell lines and an *in silico* machine learning (ML) model to predict the progression of cancer stem cell (CSC) subpopulations in colon (HCT-116) and breast (MDA-MB-231) cancer cells. Our findings indicate a significant increase in cancer stemness induced by PSNPs. Exposure to polystyrene nanoparticles stimulated the development of less differentiated subpopulations of cells within the tumor, a marker of increased tumor aggressiveness. The experimental results were further used to train an ML model that accurately predicts the development of CSC markers. Machine learning, especially genetic algorithms, may be useful in predicting the development of cancer stem cells over time.

## 1. Introduction

Cancer stem cells (CSCs) represent a group of tumor cells that have the ability to self-renew and differentiate, and can trigger tumor initiation, progression, metastasis, and recurrence [1]. CSCs show resistance to chemotherapy and radiotherapy, which contribute to tumor relapse [2]. It is known that a single CSC marker cannot fully characterize the stem-like properties of these cells. The process of identifying CSCs involves analyzing the expression of a combination of characteristic markers. In breast cancer, high expression of a cluster of differentiation 44 (CD44) and low expression of a cluster of differentiation 24 or CD24 (CD44^positive^/CD24^negative/low^) contribute to cell proliferation and tumorigenesis, while high expression of aldehyde dehydrogenase or ALDH1 is a strong indicator of metastasis [3,4]. In addition, studies have shown the functional relevance of ATP binding cassette subfamily G member 2 -ABCG2 (also known as BCRP, a breast cancer resistance protein) in relation to CSCs and therapeutic response. ABCG2 has been identified as a predictive marker of chemotherapy resistance and a potential CSC marker in solid tumors [5]. Resistance to cytotoxic agents has been attributed to the efflux of chemotherapeutic drugs by CSC-expressed ABCG2 [6]. The transmembrane glycoproteins CD44 and CD24 are potential markers for the identification of CSC populations in colon cancer [7]. High expression of CD44/CD24 cells are recognized as a subpopulation with higher clonogenic and tumor initiation potential leading to aggressive cancer types and poor prognosis [8]. During the progression from normal epithelium to adenoma, the number of ALDH1 cells increases, and they become increasingly distributed throughout the crypts [9]. Analysis of ALDH1 colon cancer stem cells (CSCs) at the molecular level showed that certain signaling pathways, including mitogen-activated protein kinases (MAPK), focal adhesion kinase (FAK), and oxidative stress survival pathways, were more active. This indicates that ALDH1 plays an important role in maintaining stemness-like properties and promoting colon tumor progression. Gaining an understanding of how these markers can predict treatment outcomes, taking into account factors such as chemoresistance, is of the utmost importance [6]. Additionally, high expression of CD44, CD24, and ALDH1 have been identified as specific markers for identifying, isolating, and tracking human colonic CSCs during the development of colorectal cancer [6]. CD44, CD24, and ALDH1 are hypothesized to be specific markers for the identification, isolation, and monitoring of human colon CSCs during colorectal cancer development. Therefore, understanding how these markers can predict treatment outcomes, especially with regard to chemoresistance, is of great importance. The global increase in plastic waste has become an issue of concern [10]. Numerous studies suggest that food or drinking water may be the source of plastic nanoparticles, which are absorbed in the intestines [6]. Vecchiotti et al. pointed out that direct contact of polystyrene nanoparticles (PSNPs) with cells causes DNA damage, via ROS production [11]. Research on *in vitro* models has shown that NP characteristics such as shape, charge, and dimensions are very important for possible toxicity [6]. Fragmentation of plastic particles in the environment leads to a higher surface-to-volume ratio, making PSNPs more reactive.

Studies have examined combined exposure to PSNPs and various drugs on fish cell lines, showing that altered pharmaceutical toxicity induced by PSNP particles may be related to incorporation rates, sorption capacity, and cellular defense mechanisms [10]. In addition, during the last few years, different mammalian *in vivo* and *in vitro* studies have been performed in order to investigate harmful effect of PSNPs (Table 1). PSNPs lead to a significant acceleration of the growth of ovarian tumors in mice, as well as to a decrease in the viability of ovarian cancer cells [12]. Xu et al. summarized 21 studies using *in vitro* Caco-2 cell models for evaluating the effects of plastic particles [13]. Domenech et al. investigated long-term effects of polystyrene nanoplastics in human colon adenocarcinoma cells (Caco-2 cells) [14]. They found that PSNPs are easily accumulated in exposed cells, and it is done in a concentration-dependent manner. In fact, at higher concentrations of PSNPs exposure, some ultrastructural alterations in mitochondria were evident, suggesting that PSNPs exposure could cause organelles’ dysfunction [14]. Importantly, internalization of NPs and MPs by normal human colon cells induces metabolic changes under both acute and chronic exposure by promoting oxidative stress, increasing glycolysis via lactate to sustain energy metabolism and glutamine metabolism to sustain anabolic processes [15]. Taken together these data provide strong evidence that NPs and MPs exposure could act as cancer risk factors for human health. Cytotoxic effects of PSNPs were also confirmed on human hepatoma HepG2 cells [16]. As an *in vitro* model of the human liver, the human hepatocellular carcinoma (HepG2) cell line was used in five recent studies [13]. PS-NPs with size of 50 nm were rapidly internalized by HepG2 cells exhibiting high negative impact on cell viability due to cellular oxidative damage and destruction of antioxidant capabilities [16]. Barguilla et al. (2022) also warn of the potential carcinogenic risk resulting from long-term exposure to micro- and nanoplastic particles, especially polystyrene nanoplastics [17]. Numerous studies warn that PSNP represents a new threat to gastric cancer and causes resistance to therapy [18]. Roje et al. (2019) indicate the potential risk of synergistic effects of chemical mixtures that include plastic nanoparticles and endocrine disrupting chemicals (EDCs) and emphasize the need for a more precise definition of an action plan for the management of risks from EDCs and plastic waste at the global level [19]. Oral administration of polyethylene nanoplastics was found to significantly affect the intestinal microenvironment in mice. This disruption of the microenvironment favors the development of colorectal tumors due to changes in the adaptive immune response [20]. The combined toxicity of micro- and nanoplastics causes serious damage to the intestinal barrier. Considering that most studies on PS micro- and nanoplastics so far only investigate one particle size, it is possible that the health risks associated with exposure to PS micro- and nanoplastics in the body are underestimated [21].

Our study aimed to investigate the relationship between the expression levels of CSC markers and chemosensitivity, as well as to analyze the effect of PSNP on the expression patterns of these markers. Our goal was to better understand the basic molecular mechanisms in cancer cells. To achieve this, we used a machine learning (ML) model, specifically a genetic algorithm (GA), to improve our understanding and prediction of mechanisms in CSC development. Computer modeling and simulation in the field of science has become essential. Computer models enable fast, easy, and cost-effective simulation of complex, time-consuming and expensive experiments. Machine learning (ML) models are designed to mimic real processes. In this study, we used genetic algorithms (GA) as a metaheuristic method to generate high-quality solutions to optimization and search problems, drawing inspiration from Charles Darwin’s theory of natural evolution [22]. GA uses mathematical operators such as mutation, crossover, and selection, inspired by biological processes, to optimize solutions [23]. Our study significantly contributes to the application of artificial intelligence (AI) methods for more efficient analysis of biomedical data, particularly focusing on the use of cancer stem markers for personalized prediction purposes. Specifically, we analyzed the effect of PSNPs on stem-like characteristics of colon and breast cancer cells. Examination of CSC markers was performed using flow cytometry analysis. The resulting experimental data were then used to develop and validate a machine learning/genetic algorithm (ML/GA) model, with the goal of improving the prediction of cancer outcomes over time. Investigating the effect of PSNPs on the cancer stem and analyzing the expression of CSC markers aims to gain a more detailed insight into the complex dynamics of cancer. This knowledge will allow us to optimize treatment strategies by tailoring them to the specific needs of individual patients, taking into account their personalized information. The goal of this research is to improve patient outcomes and contribute to the advancement of cancer treatment.

## 2. Materials and Methods

### 2.1. Data Study

The use of machine learning and genetic algorithms in the processing of biomedical data is still not sufficiently exploited. Therefore, before all experiments, we performed a very extensive analysis of the available literature by using the Google Scholar platform to obtain statistical data related to the topics of cancer stem cells analysis and the use of ML and GA. Only keywords are included, individually or in combinations. All listed calculations are presented in Figures S1 and S2 in Supplementary Materials.

### 2.2. Cell Cultures and Polystyrene Particles Treatment

Human colorectal carcinoma HCT-116 cell line and a human breast cancer MDA-MB-231 cell line (purchased from the European Collection of Authenticated Cell Cultures—ECACC, London, UK) were cultured in Dulbecco’s Modified Eagle Medium (DMEM) (D5796; Sigma–Aldrich Chemical Company, St. Louis, MO, USA) supplemented with 10% fatal bovine serum (F4135-500ML; Sigma–Aldrich Chemical Company, St. Louis, MO, USA) and 1% penicillin/streptomycin (P4333-100ML; Sigma–Aldrich Chemical Company, St. Louis, MO, USA). Both cell cultures grew in 75 cm^2^ culture flasks and were maintained in a humidified atmosphere with 5% CO_2_ at a physiological temperature of 37 °C. The media were changed every 2 days and cells were trypsinized when necessary (0.05% trypsin–0.53 mM EDTA). After a few passages and a confluence of about 80%, the human colorectal carcinoma cells and breast cancer cells were treated with medium containing PS nanoparticles (2.2 × 10^10^ PSNPs/mL). The polystyrene particles used in the experiments were carboxylate-modified 40 nm (red 8793 Thermo Fisher, Waltham, MA, USA) PS-fluospheresTM. Prior to each cell culture experiment, stock solutions of PS particle were prepared as previously described [10]. After treatment incubation of 24 h, 33 h, 43 h, 52 h, and 76 h, the cells were harvested for flow cytometry analysis or cytotoxicity assay.

### 2.3. Flow Cytometry Analysis

Flow cytometry was performed following routine procedures by using 1 × 10^6^ cells per sample, and by using fluorochrome-labelled anti-mouse mAb specific for CD24, ALDH1 or isotype-matched control (BD Biosciences, San Jose, CA, USA). For intracellular staining, cells were fixed in Cytofix/Cytoperm, permeated with 0.1% saponin, and stained with fluorochrome-labelled anti-human mAb specific for ABCG2 (BD Biosciences, San Jose, CA, USA). Control cultures of cells without treatment were also included in all experiments. Flow cytometry was conducted on FACSCalibur Flow Cytometer (BD Biosciences, San Jose, CA, USA) and the data was analyzed using the Flowing software analysis (2.5.1.Turku Bioscience, Turku, Finland). 

### 2.4. Machine Learning Model (ML)—Genetic Algorithm (GA)

The GA symbolic regressor can provide a symbolic mathematical function that most accurately represents the input data. The output is usually intelligible, and easily transferable to another application or environment. This is GA’s strongest quality. In the GA, the mathematical function is represented as a tree, with the sheets serving as the variables or constants and the functions serving as the nodes (branching points). Nodes have the possibility to be different functions from the list -function set [add, sub, mult, div, sqrt, log, abs, neg, inv, max, min, sin, cos, tan], leaves -determined in the terminal set for constant values of defined range or variables. Nodes and leaves are primarily acquired randomly; crossover and mutation reproduction change them. Following the execution of the genetic operation, the population of children is examined to determine the effectiveness of the results and to choose the best outcomes through a tournament selection that will take part in the following iteration of the genetic algorithm. Once the algorithm has reached the stopping threshold or the maximum number of generations, the loop is stopped. The operating principles are detailed and described by O’Neill et al. [24]. GA is not sequential or time-dependent and does not have memory. It is a simple algorithm that sets the past input values of time series in multiple points and other variables for the prediction of future value. In this paper, PyGAD, a Python Genetic Algorithm library, played a pivotal role in conducting Genetic Algorithm (GA) experiments. Developed by Ahmed Fawzy Gad [25], PyGAD stands out for its intuitive interface and efficient implementation, enabling seamless integration into the research workflow. Leveraging PyGAD, the study harnessed the power of Genetic Algorithms to explore and optimize complex problem spaces. With its diverse functionality and robust performance, PyGAD facilitated the fine-tuning of algorithm parameters, model training, and result analysis, thus contributing significantly to the advancement of the research objectives. The utilization of PyGAD underscored the importance of accessible and user-friendly tools in enabling researchers to harness the potential of Genetic Algorithms for solving real-world problems efficiently.

Input data for training GA and fitting was used from experimental measurements of CSC markers. The cancer cells were treated with PSNPs, while stem markers were followed by flow cytometry. Several markers (in both cell lines: ABCG2, ALDH, CD24, CD24ABCG2, CD24ALDH) were measured in time-dependent manner (24 h, 33 h, 43 h, 52 h). Results from measurements at 76 h were used as blind data for GA model validation. The objective was to develop a Genetic Algorithm (GA) capable of predicting future outcomes based on input data. Experimental measurements collected at time points 24 h, 33 h, 43 h, and 52 h were utilized to construct an optimal GA curve, which was then employed to forecast values at 72 h and 96 h time points. To assess accuracy, a real experimental measurement was conducted at the 72-h mark, and the disparity between the GA prediction and the actual measurement was evaluated. This study exclusively focuses on GA model, without conducting a comparative analysis with other similar machine learning methods. Several factors contribute to this decision. Firstly, the research aims to assess the effectiveness and applicability of GA within the specific problem domain under investigation. By concentrating solely on GA, the study seeks to thoroughly investigate its capabilities, strengths, and limitations without the potential complexities introduced by comparing multiple methods. GA excels in optimizing problems characterized by complex and poorly understood search spaces. While methods like logistic regression or decision trees may offer greater interpretability and ease of implementation, they often require substantial amounts of input data to yield satisfactory results. Furthermore, the authors chose GA because it is renowned for its proficiency in handling large search spaces and tackling non-linear optimization problems.

The coefficient of determination *R*^2^ is used to assess the obtained model. It reflects how well the statistical model fits the data under investigation. It is the proportion of variance in the dependent variable that is explained by the model.

$$R^{2}=1-\frac{SSR}{SST}$$

where: *SSR* is a Sum of Squared Regression (variation explained by model), and *SST* is Sum of Squared Total (total variation in data) [26].

### 2.5. Statistical Analysis

In this study, we utilized Statistical Package for the Social Sciences v23.0 software IBM Coro., Armonk, NY, USA (SPSS Inc.) For each biomedical analysis, three individual experiments were executed with a minimum of three replicates, unless stated otherwise. The data are presented as means with standard deviation (SD). Statistical analyses were performed using Mann–Whitney test and one-way analysis of variance (ANOVA).

## 3. Results

### 3.1. CSC Protein Marker Analyses—Flow Cytometry

**Figure 1:**
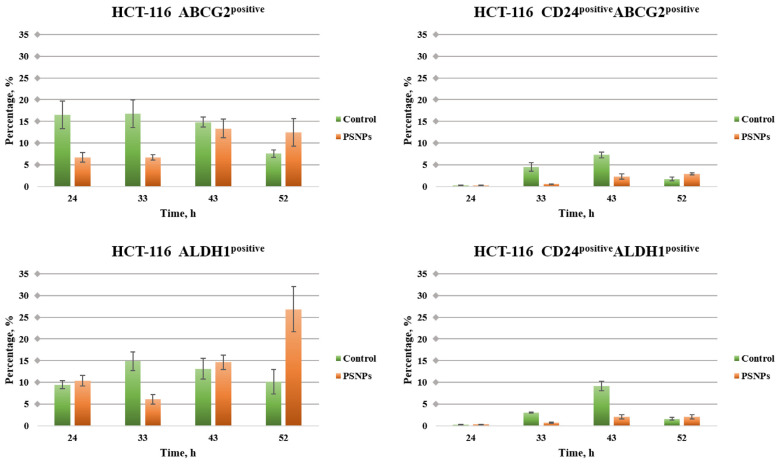
The effects of PSNPs on the expression rate of CSC markers in HCT-116 cells. Expression rate of untreated as well as PSNPs-treated cells, analyzed by flow cytometry. The data are presented as means ± SEM of three independent experiments.

PSNPs in the early periods of incubation during the treatment of HCT-116 cells induce a decrease, while in the later periods (43 and 52 h from treatment) they induce an increase in the ABCG2 marker (Figure 1). A trend of significant increase in the ABCG2 expression with time is observed. The same is observed with the ALDH1 marker, which steadily increases over time. After 52 h of treatment, the expression of the ALDH1 marker, an indicator of metastasis, is higher than in control cells by about 2.5-fold. The CD24^positive^/ABCG2^positive^ subpopulation in HCT-116 cells also grows steadily and significantly over time. Up to 43 h from treatment, the detected subpopulation is higher in control cells, while after 52 h this subpopulation is significantly more represented in PSNP-treated cells. The CD24^positive^/ALDH1^positive^ subpopulation in PSNP treatment increases in the first periods of treatment, but in the later periods this population stabilizes and is generally not significantly more expressed than in control cells.

**Figure 2:**
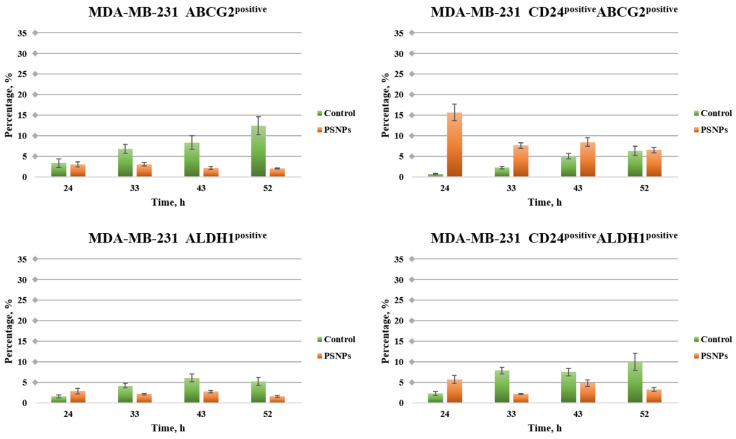
The effects of PSNPs on the expression rate of CSC markers in MDA-MB-231 cells. Expression rate of untreated as well as PSNPs-treated cells, analyzed by flow cytometry. The data are presented as means ± SEM of three independent experiments.

In MDA-MB-231 cells, in contrast to HCT-116 cells, we observed in the PSNP treatment that the ABCG2 marker decreases over time and that it is significantly lower than in control cells at all times (Figure 2). The same applies to the expression of the ALDH1 marker, which also decreases over time and is statistically significantly lower than in control cells. Again, in contrast to HCT-116 cells, in MDA-MB-231 cells the CD24^positive^/ABCG2^positive^ subpopulation has the highest acute effect (i.e., after 24 h of treatment). In the later periods of incubation, this subpopulation is significantly less but stably represented and expressed more than in the control cells. The CD24^positive^/ALDH1^positive^ subpopulation in the PSNP treatment in the MDA-MB-231 cells was most significantly expressed at the beginning of treatment (24 h), while later it steadily decreased and was less expressed than in control cells. The proportion of cell marker expression in HCT-116 and MDA-Mb-231 cell populations is presented in Table S1 in the Supplementary Materials.

### 3.2. ML Model

**Figure 3:**
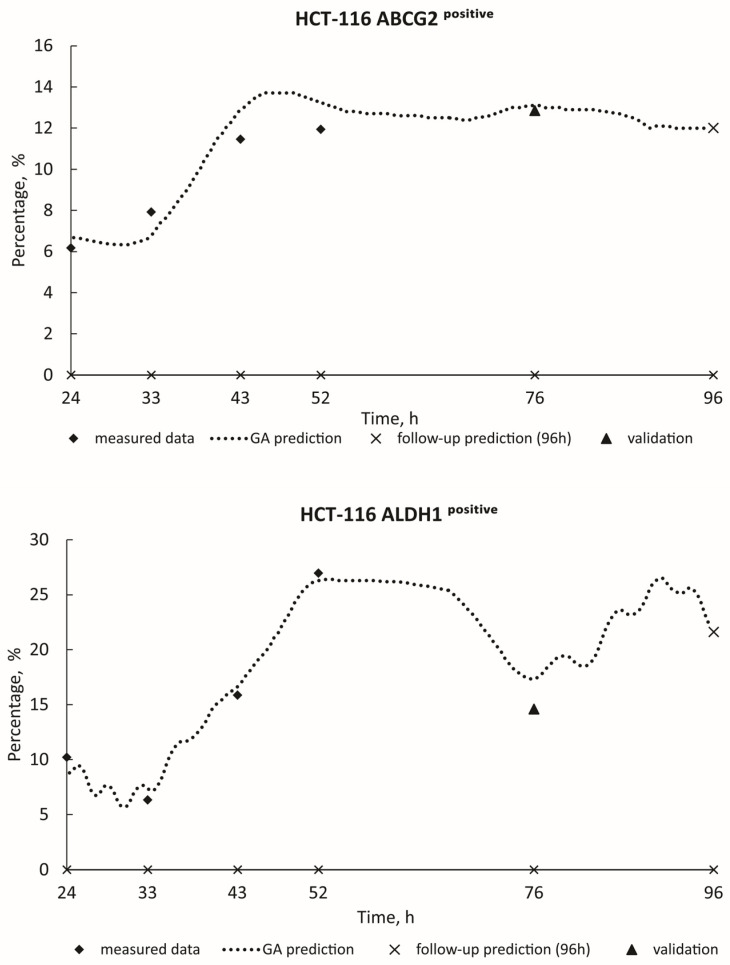
GA prediction of HCT-116 cell growth in the PSNP treatment: ABCG2^positive^ (GA decision tree was present on Figure S3); ALDH1^positive^ (GA decision tree was present on Figure S4).

**Figure 4:**
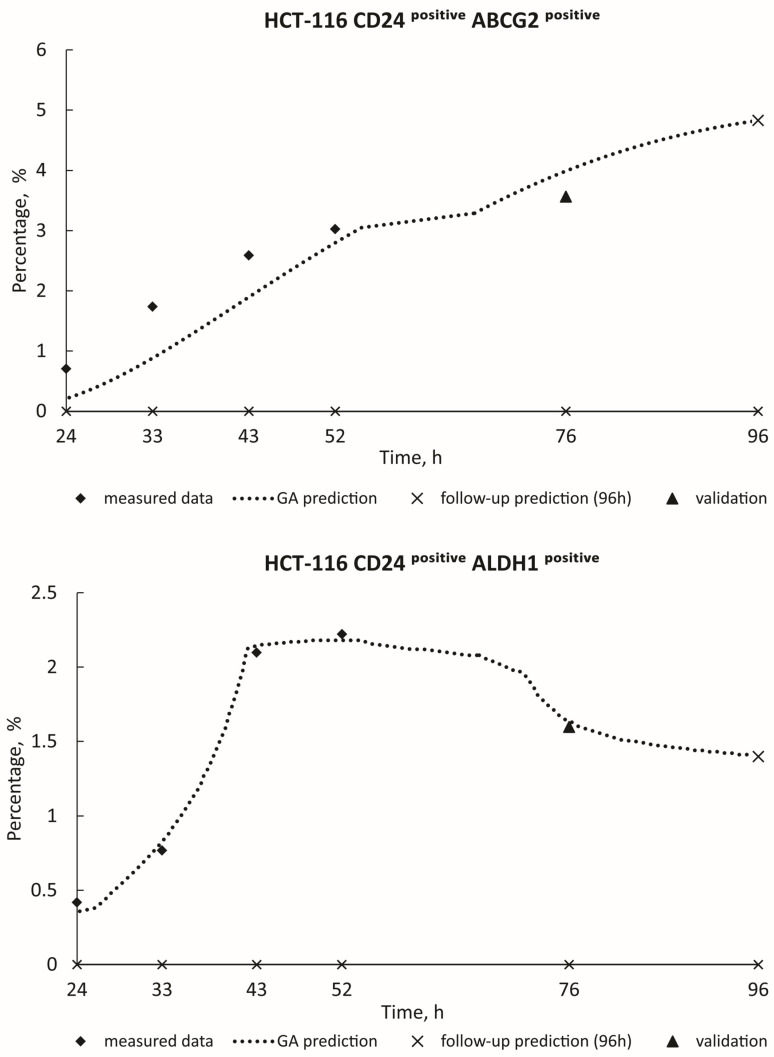
GA prediction of HCT-116 cell growth in the PSNP treatment: CD24^positive^ABCG2^positive^ (GA decision tree was present on Figure S5); CD24^positive^ ALDH1^positive^ (GA decision tree was present on Figure S6).

**Figure 5:**
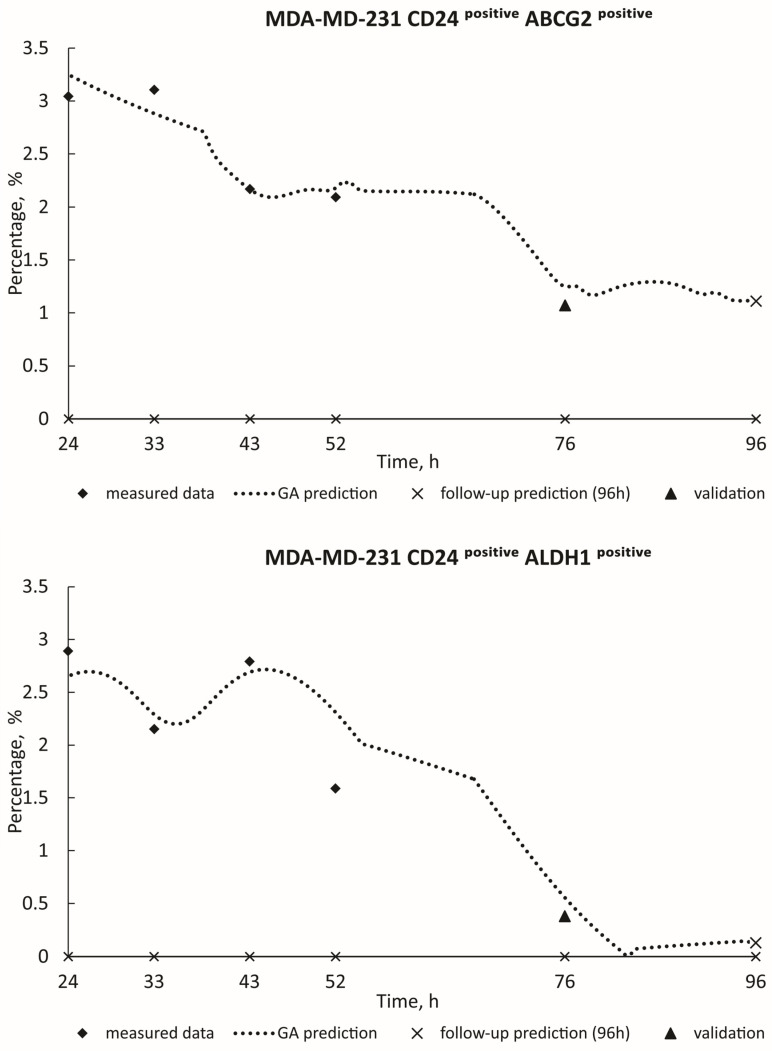
GA prediction of MDA-MB-231 cell growth in the PSNP treatment: CD24^positive^ ABCG2^positive^ (GA decision tree was present on Figure S7). CD24^positive^ ALDH1^positive^ (GA decision tree was present on Figure S8).

**Figure 6:**
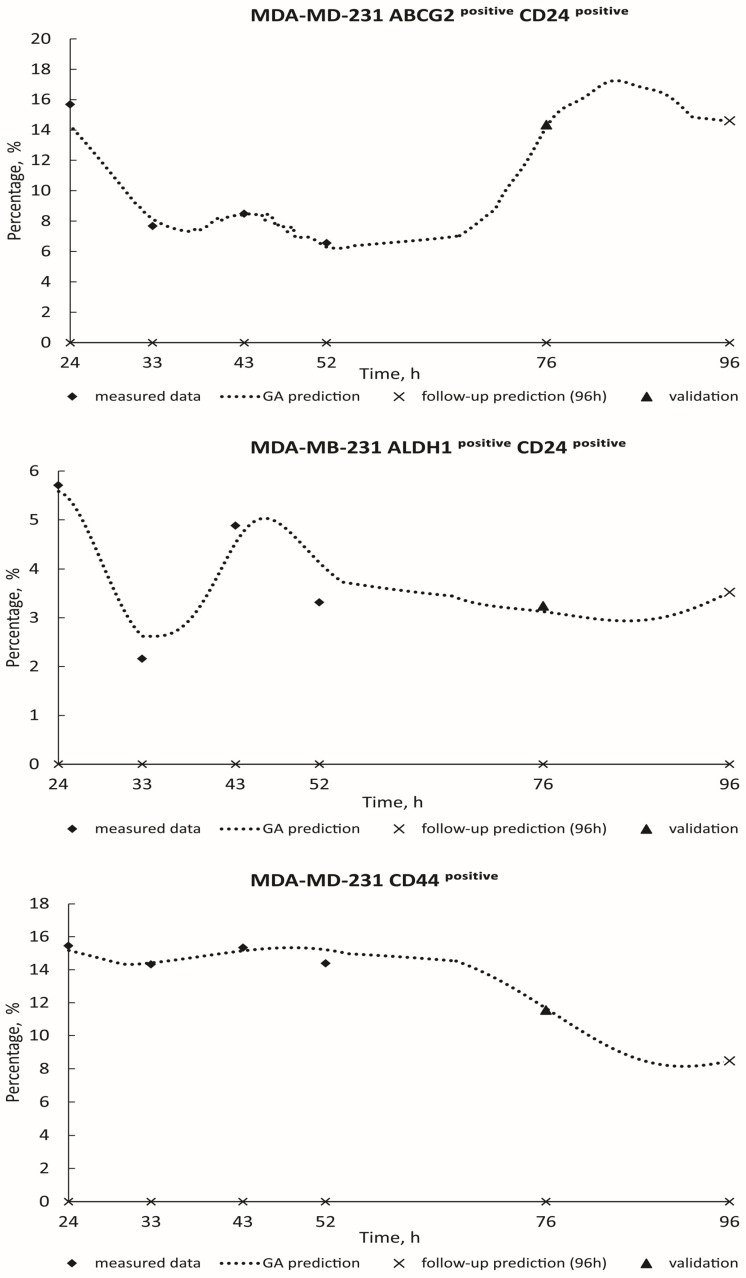
GA prediction of MDA-MB-231 cell growth in the PSNP treatment: ABCG2^positive^ CD24^positive^ (GA decision tree was present on Figure S9). ALDH1^positive^ CD24^positive^ (GA decision tree was present on Figure S10). CD44^positive^ (GA decision tree was present on Figure S11).

Figure 3 and Figure 4 show the real measured data for HCT-116 (ABCG2^positive^; ALDH1^positive^; CD24^positive^ ABCG2^positive^; CD24^positive^ ALDH1^positive^) from 24 h to 52 h (represented by diamond dots), alongside the estimation of deceased cases (illustrated by dashed curves) spanning from 24 h to 96 h. The measured blind data at 76 h (indicated by triangle dots) were utilized for the validation of the GA decision model, with follow-up predictions for the 96-h mark presented as X dots on the graphics. Algorithm estimate scores with corresponding *R*^2^ values are provided in Table 2. Additionally, Figure 5 and Figure 6 show the real measured data for MDA-MB-231 (CD24^positive^ ABCG2^positive^; CD24^positive^ ALDH1^positive^; ABCG2^positive^ CD24^positive^; ALDH1^positive^ CD24^positive^; CD44^positive^) (represented by dots) and the estimation of deceased cases (depicted by orange curves) spanning from 24 h to 96 h. The measured blind data at 76 h (indicated by triangle dots) were utilized for the validation of the GA decision model, with follow-up predictions for the 96-h mark presented as X dots on the graphics. Table 2 similarly presents the algorithm estimate scores along with their respective *R*^2^ values. When the coefficient of determination *R*^2^ approaches unity, it signifies a high level of correlation between the predicted values generated by the model and the actual observed data. In this paper, the remarkable closeness of *R*^2^ values to 1 across various experimental conditions underscores the robustness and accuracy of the predictive models developed. Such high *R*^2^ scores indicate that the models adeptly capture the underlying patterns and relationships within the data, thereby enabling precise forecasts of future outcomes. These findings not only validate the efficacy of the employed methodologies but also instill confidence in the reliability of the predictive models. Moreover, the proximity of *R*^2^ to 1 suggests that the models exhibit minimal error in their predictions, making them valuable tools for decision-making and planning in real-world scenarios.

## 4. Discussion

It is known that plastic is everywhere around us. It is estimated that about 5.25 trillion plastic particles are present in the oceans alone, which poses a danger to living organisms, including humans [27,28,29]. The effects of nanoplastics are often chronic. We currently know very little about them, but research shows that increasing the concentration of nanoplastics enhances inflammatory, cytotoxic, and genotoxic effects. More toxicological research is needed to better understand the negative effects of nanoplastics on the environment and humans. It is important to expand research in this area in order to find a solution to the pollution problem that arises from it [11]. Studies investigating the impact of polystyrene nanoparticles on a subpopulation of cancer stem cells consider the presence of polystyrene nanoparticles in the everyday environment as a major pollutant [30]. The use of a genetic algorithm and machine learning model approach in the analysis of cancer stem cell markers is an unexplored area of research. In this sense, we are convinced that the combination of *in silico* and *in vitro* studies is a good modeling system in the treatment of biomedical markers of cancer stem cells. The effect of PSNPs on cancer stem cells is also an underexplored area. We investigated the expression patterns of the markers in HCT-116 cells treated with PSNPs. The proportion of cells expressing the marker ABCG2 ranged from 6.71% to 13.39% in the entire cell population. Likewise, the ALDH1^positive^ subpopulation ranged from 6.13% to 26.8% within the HCT-116 cell population. We also examined the CD24^positive^/ABCG2^positive^ subpopulation, which accounted for 0.31% to 2.94% of the HCT-116 cell population. Similarly, the CD24^positive^/ALDH1^positive^ subpopulation accounted for 0.34% to 2.09% of the entire HCT-116 cell population. These findings provide valuable insight into the distribution and proportions of specific marker subpopulations in the HCT-116 cell population treated with PSNP. These observations add to the understanding of the effects of PSNPs on the expression patterns of these markers in cancer cells, highlighting the potential influence of PSNPs on cancer stemness and the development of targeted treatment strategies [30]. Investigation of a subpopulation of MDA-MB-231 cells treated with polystyrene nanoparticles (PSNPs) revealed interesting findings regarding the expression of specific markers. The percentage of cells expressing the marker ABCG2 ranged from 2.09% to 3.11% within the MDA-MB-231 population. Similarly, the ALDH1 subpopulation was represented in 1.59% to 2.89% of the total MDA-MB-231 cell population. These observations indicate the presence of distinct subpopulations within the MDA-MB-231 cell line. In addition, analysis of the CD24^positive^/ABCG2^positive^ subpopulation showed a range from 6.56% to 15.69% within the MDA-MB-231 cell population. The CD24^positive^/ALDH1^positive^ subpopulation ranged from 2.16% to 5.71% of the total MDA-MB-231 cell population. These specific marker subpopulations provide insight into the distribution and proportions of cells that have increased malignant potential after PSNP treatment. Sulukan et al. have shown in their study the effect of PSNP on various biological processes associated with cancer using a zebrafish model. Their findings indicated significant effects of nanosized polystyrene particles on cancer-related mechanisms [31]. Understanding the dynamics and proportions of these marker subpopulations is critical for assessing the impact of PSNPs on cancer stemness marker expression. These nanoparticles are able to suppress CSCs and may contribute to tumor progression. Previous studies have shown that ABCG2 and human clusters of differentiation 33 (CD133) markers are highly expressed in drug-resistant and highly tumorigenic cell lines, such as MDA-MB-231 and MCF-7, which resemble cancer stem cells (CSCs) [32]. Although the CD24^positive^/ABCG2^positive^ and CD24^positive^/ALDH1^positive^ subpopulations are less represented in the whole cell populations, these combinations indicate increased aggressiveness of cancer cells in terms of progressivity and drug resistance potential [33]. The differential expression of ABCG2 and ALDH1 markers between HCT-116 cells and MDA-MB-231 breast cancer cells indicates different characteristics and behavior of these cell lines. The higher expression of ABCG2 and ALDH1 in HCT-116 cells suggests a potential role in drug resistance and stem-like properties specific to colorectal cancer. On the other hand, MDA-MB-231 cells, which are of metastatic origin, show different characteristics compared to HCT-116 cells. Metastatic cancer cells can spread from the primary tumor to distant sites in the body, leading to a more aggressive and invasive phenotype. The metastatic nature of MDA-MB-231 cells may explain the higher expression of aggressive subpopulations, such as CD24^positive^/ABCG2^positive^ and CD24^positive^/ALDH1^positive^ cells [34]. These subpopulations are associated with increased tumorigenic potential and resistance to conventional treatments. Acquisition of metastatic properties by MDA-MB-231 cells contributes to their aggressive behavior and ability to colonize new tissues [35]. MDA-MB-231 cells are known for their increased aggressiveness and metastatic potential. In contrast, HCT-116 cells show moderate invasiveness and reduced metastatic capacity. HCT-116 cells are derived from the primary tumor and may have different molecular characteristics compared to MDA-MB-231 cells. The primary tumor environment differs from that of distant metastases, and cells originating from these different stages of cancer progression show different expression patterns of markers such as ABCG2 and ALDH1. The differential expression of ABCG2 and ALDH1 markers between these cell lines highlights the heterogeneity and complexity of cancer. By examining the individual characteristics of each cancer cell, insight is gained into the basic mechanisms that drive tumor progression and metastasis, which ultimately leads to the development of specific targeted therapies that correspond to certain types and stages of cancer. In this study, a GA algorithm was used to develop a model that would have the ability to estimate the growth of PSNP-treated cells over time. The model was trained to predict the behavior of the cells in the future time at 76 h and 96 h. Each individual prediction model has an archived high accuracy with a high coefficient of determination *R*^2^. The average *R*^2^ was 0.979 (min. 0.93–max. 0.99) for the 76-h prediction. Based on these results, we can conclude that GA can be used as a very precise auxiliary tool for *in silico* testing, analysis, and monitoring of cancer stem cell subpopulation behavior. The advantage of such models is that they allow us to precisely monitor the state of the cell’s behavior at any moment, in contrast to experimental measurements that are discretized in time intervals.

## 5. Conclusions

In conclusion, this study highlights several key strengths. Using machine learning, especially genetic algorithms, it is possible to accurately model and predict the development of cancer stem cells over time. Investigating the effect of PSNPs on the cancer stem and analyzing the expression of CSC markers aims to gain a more detailed insight into the complex dynamics of cancer, as well as the potential effects of environmental pollution on cancer. Polystyrene nanoparticles stimulated the development of less differentiated cell subpopulations within the tumor, thereby increasing the level of biological aggressiveness of the tumor. Validation Machine learning as a reliable and useful approach is recommended for analyzing large biomedical databases. Research like this improves our understanding of cancer stem cells. In this way, the outcome of patient treatment is improved and contributes to the improvement of cancer therapy.

## Figures and Tables

**Table 1:** Summary of *in vitro* and *in vivo* studies using polystyrene particles.

Biological Models	Plastic Particle Source	Polymer Type	Particle Size	Exposure Concentration	Results
*In vivo*: epithelial ovarian cancer mice model [12]	Purchased from Huge Biotechnology (Shanghai, China)	polystyrene	100 nm	10 mg/L for 27 days	PS-NP exposure accelerated EOC tumor growth in mice
*In vitro*: human colon adenocarcinoma cells (Caco-2) [14]	Commercially obtained (Spherotech, Inc., Chicago, IL, USA)	polystyrene	50 nm	range of different concentrations: 0, 6.5, 13, 26, and 39 μg/cm^2^	Accumulation of PSNPs in exposed cells in a concentration-dependent manner
*In vitro*: normal human intestinal cells (CCD-18Co) [15]	purchased from Sigma–Aldrich (St Louis, MO, USA)	polystyrene	0.5 μm and 2 μm	range of different concentrations (1–5-10–20 μg/mL)	NPs and MPs exposure cause oxidative stress
*In vitro*: HepG2 cells [16]	obtained from the DK Nano Tech (Beijing, China)	polystyrene	50 nm	10 μg/mL for 12 h	reduced the cell viability
*In vitro*: mouse embryonic fibroblasts [17]	purchased from Spherotech (Chicago, IL, USA)	polystyrene	50 nm	increasing doses of PSNPLs (10, 25, 75, and 100 μg/mL) for 24 h	exacerbated cancer
*In vivo*: BALB/c nude mice *In vitro*: human gastric cancer cell lines (AGS, MKN1, MKN45, NCI-N87, and KATOIII) [18]	purchased from Cospheric (Somis, CA, USA)	polystyrene	9.5–11.5 µm	*In vivo*: 1.72 × 10^4^ particles/mL *In vitro:* 8.61 × 10^5^ particles/mL	induced resistance to chemo- and monoclonal antibody-therapy
*In vitro*: human breast cancer cell lines: MDA-MB 231, and MCF-7 [19]	purchased from Thermo Fisher Scientific, Waltham, MA, USA	polystyrene	60 nm	1, 10, and 100 mg/mL	influence cell viability and proliferation
*In vivo:* C57BL/6 J mice [20]	purchased from Magsphere (Pasadena, CA, USA)	polyethylene	50.7, 503.6, and 5047.0 nm	20 mL/kg body weight, for 28 consecutive days	causing severe dysfunction of the intestinal barrier

**Table 2:** Score of the prediction.

Model System	*R*^2^—Score of the Prediction
HCT-116 ABCG2^positive^	0.99968
HCT-116 ALDH1^positive^	0.98868
HCT-116 CD24^positive^ ABCG2^positive^	0.95683
HCT-116 CD24^positive^ ALDH^positive^	0.99745
MDA-MB-231 CD24^positive^ ABCG2^positive^	0.96353
MDA-MB-231 CD24^positive^ ALDH1^positive^	0.95011
MDA-MB-231 ABCG2^positive^ CD24^positive^	0.99847
MDA-MB-231 ALDH1^positive^ CD24^positive^	0.93221
MDA-MB-231 CD44^positive^	0.99055

## Data Availability

The data presented in this study are available in this article and Supplementary Materials.

## Supplementary Materials

The following supporting information can be downloaded at: https://www.mdpi.com/article/10.3390/toxics12050354/s1, Figure S1: Data study for Cancer Stem Cells, Polystyrene nanoparticles, Machine learning model, Genetic algorithm, HCT-116, and MDA-MB-231. Source Google Scholar 2012–2022. Figure S2: Data study for CD24, CD44, ALDH1, and ABCG2. Source Google Scholar 2012–2022. Figure S3: GA decision tree for HCT-116 cell growing in PSNP treatment–ABCG2^positive^. Figure S4: GA decision tree for HCT-116 cell growing in PSNP treatment–ALDH1^positive^. Figure S5: GA decision tree for HCT-116 cell growing in PSNP treatment–CD24^positive^ABCG2^positive^. Figure S6: GA decision tree for HCT-116 cell growing in PSNP treatment–CD24^positive^ ALDH1^positive^. Figure S7: GA decision tree for MDA-MB-231 cell growing in PSNP treatment–CD24^positive^ABCG2^positive^. Figure S8: GA decision tree for MDA-MB-231 cell growing in PSNP treatment–CD24^positive^ALDH1^positive^. Figure S9: GA decision tree for MDA-MB-231 cell growing in PSNP treatment–ABCG2^positive^CD24^positive^. Figure S10: GA decision tree for MDA-MB-231 cell growing in PSNP treatment–ALDH1^positive^CD24^positive^. Figure S11: GA decision tree for MDA-MB-231 cell growing in PSNP treatment–CD44^positive^. Table S1: Proportion of cell marker expression in HCT-116 and MDA-Mb-231 cell populations.
