# Supplementary material for: Machine Learning Model for Prediction of Development of Cancer Stem Cell Subpopulation in Tumurs Subjected to Polystyrene Nanoparticles
Source: Toxics. 2024 May 10;12(5):354. doi: 10.3390/toxics12050354 (PMC11125870; doi:10.3390/toxics12050354)
Supplement: Supplementary file 1 [file toxics-12-00354-s001.zip › Table S1.pdf]

Table S1: Proportion of cell marker expression in HCT-116 and MDA-Mb-231 cell populations

**HCT-116 treated with PSNP**	**Ranged from 24 h to 52 h**
**ABCG2+**	6.71% - 13.39%
**ALDH1+**	6.13% - 26.8%
**CD24+/ABCG2+**	0.31% - 2.94%
**CD24+/ALDH1+**	0.34% - 2.09%
**MDA-MB-231 treated with PSNP**	**Ranged** from 24 h to 52 h
**ABCG2+**	2.09% - 3.11%
**ALDH1**	1.59% - 2.89%
**CD24+/ABCG2+**	6.56% - 15.69%
**CD24+/ALDH1+**	2.16% - 5.71%
