# Supplementary figures and images for: Machine Learning Model for Prediction of Development of Cancer Stem Cell Subpopulation in Tumurs Subjected to Polystyrene Nanoparticles
Source: Toxics. 2024 May 10;12(5):354. doi: 10.3390/toxics12050354 (PMC11125870; doi:10.3390/toxics12050354)

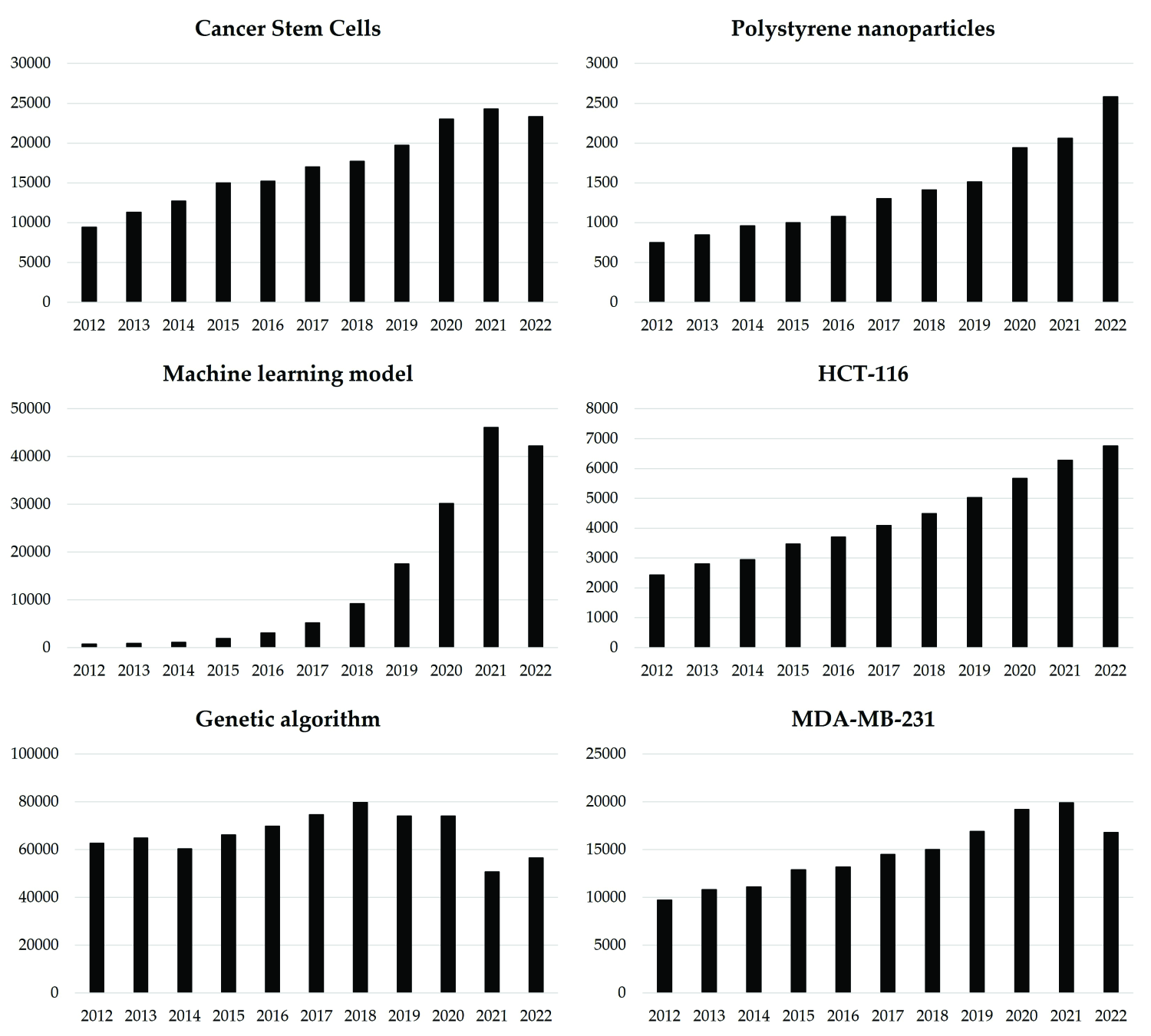

Supplement: Supplementary file 1 [file toxics-12-00354-s001.zip › Figure S01.tif]

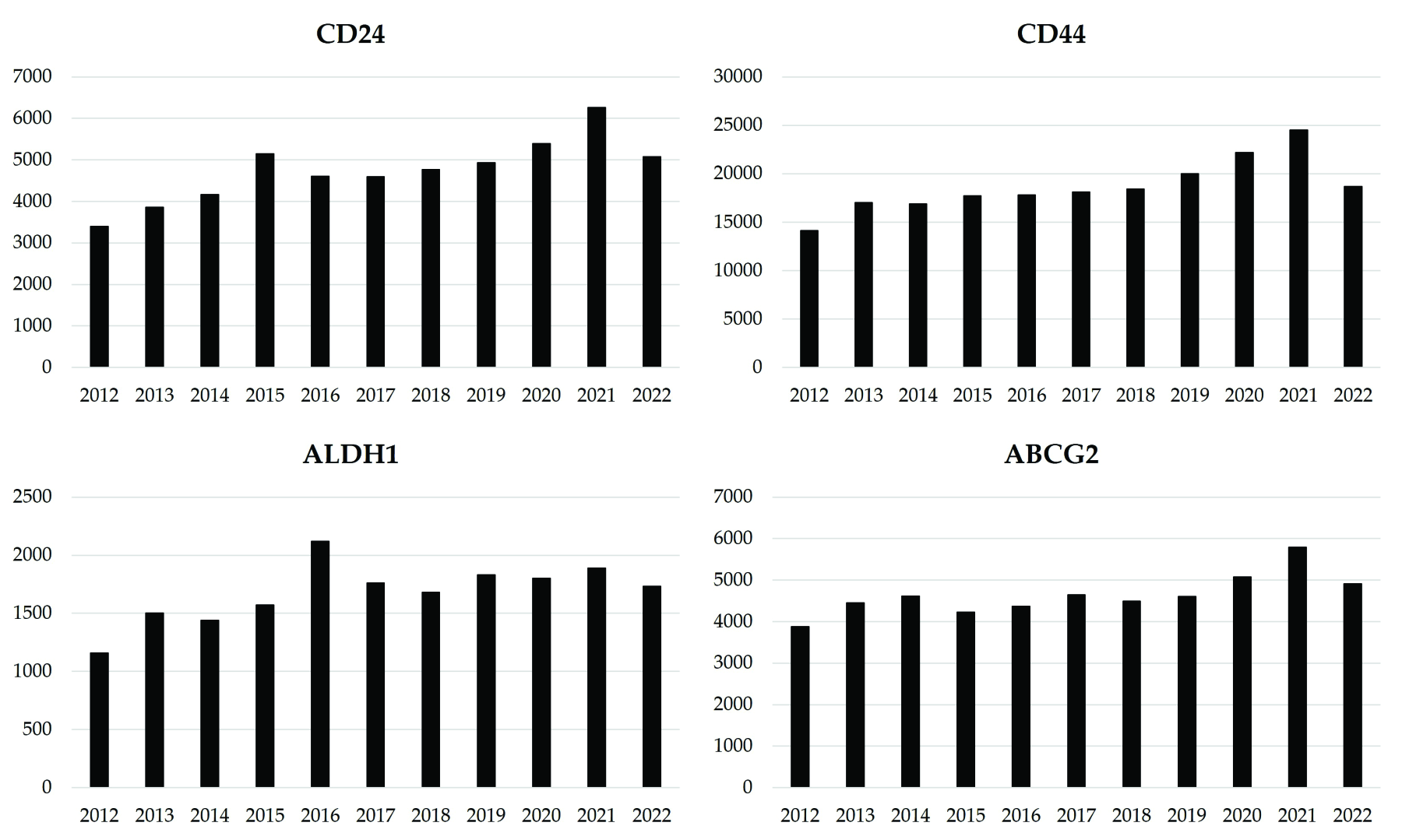

Supplement: Supplementary file 1 [file toxics-12-00354-s001.zip › Figure S02.tif]

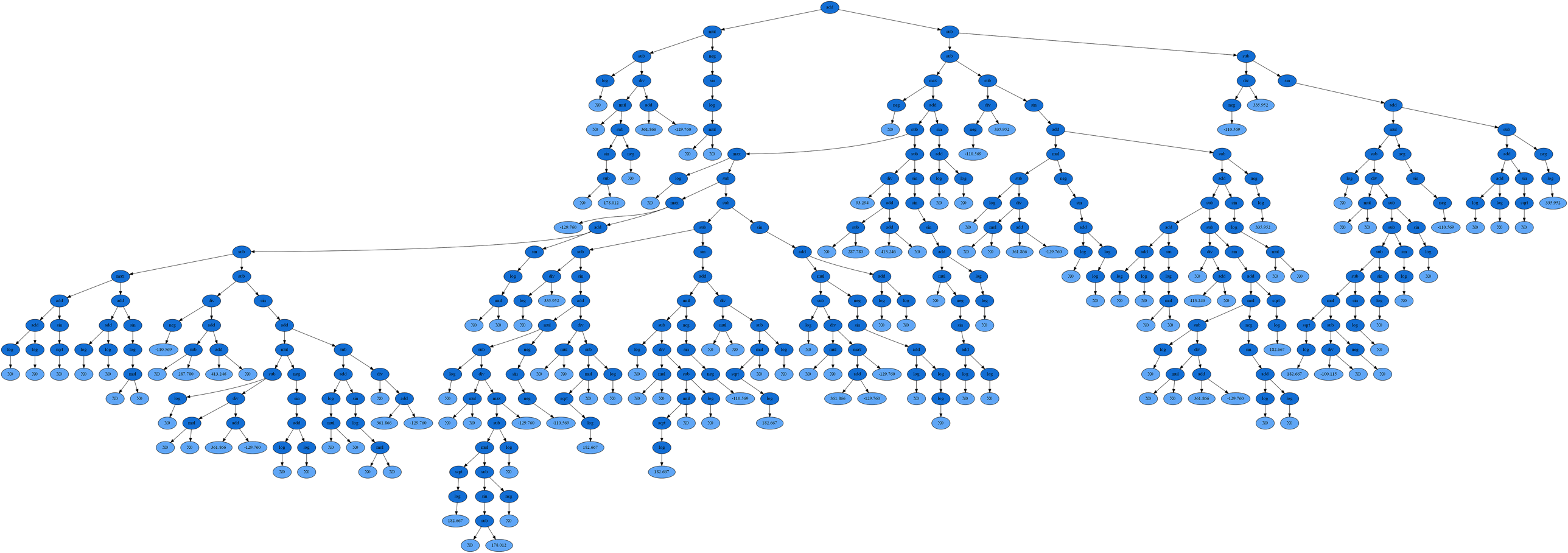

Supplement: Supplementary file 1 [file toxics-12-00354-s001.zip › Figure S04.tif]

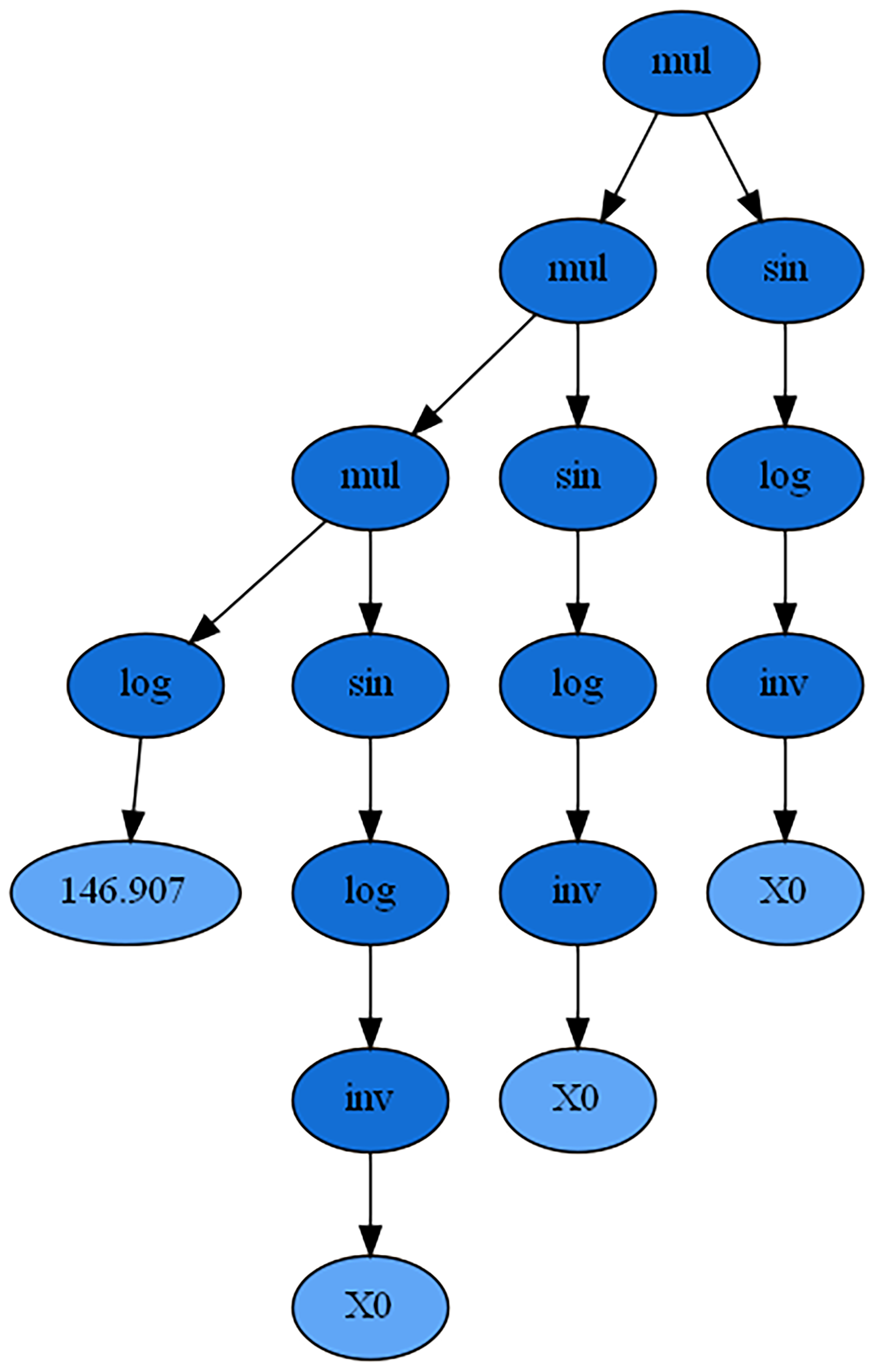

Supplement: Supplementary file 1 [file toxics-12-00354-s001.zip › Figure S05.tif]

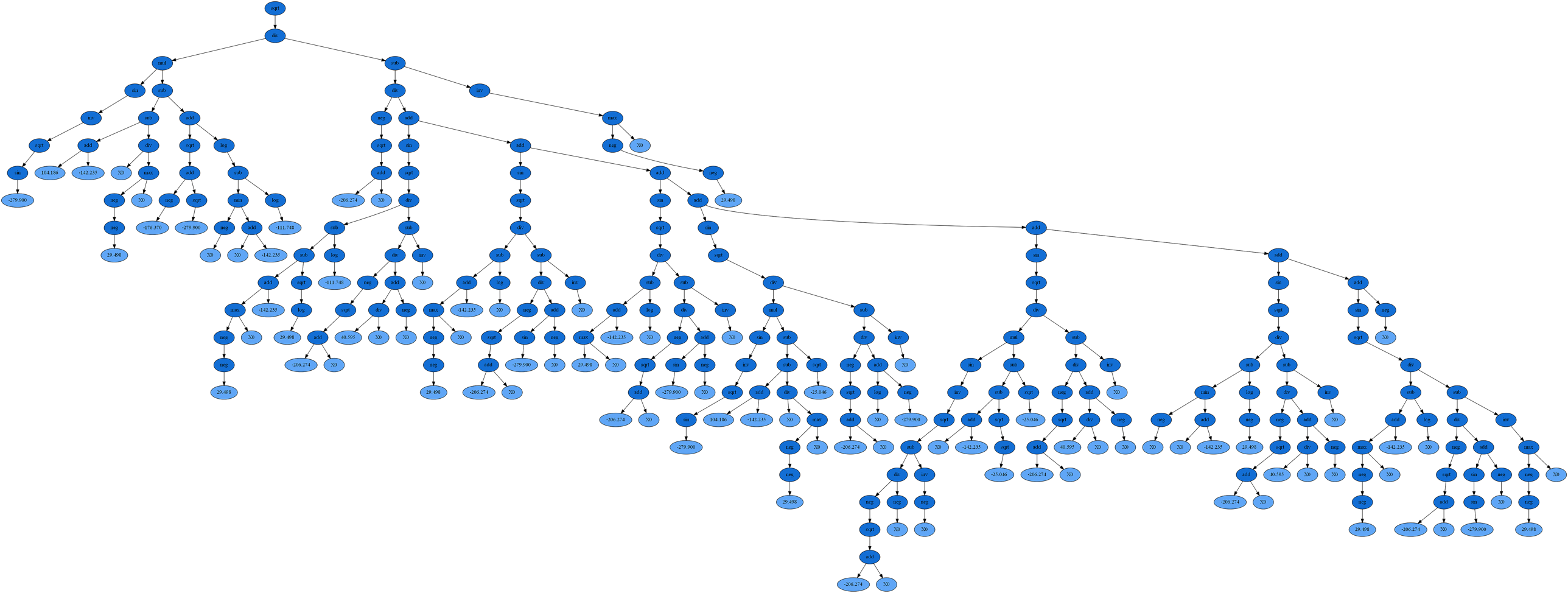

Supplement: Supplementary file 1 [file toxics-12-00354-s001.zip › Figure S06.tif]

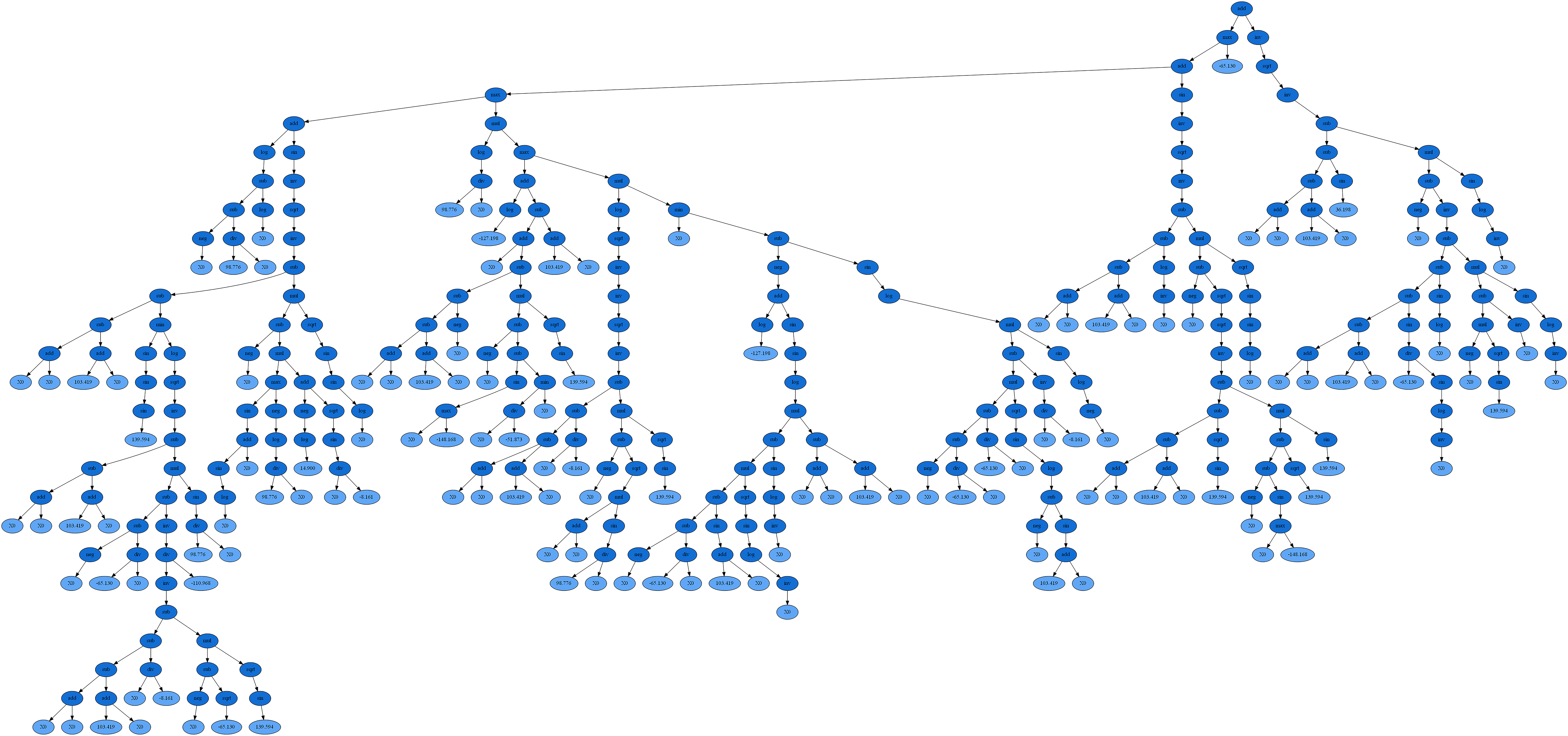

Supplement: Supplementary file 1 [file toxics-12-00354-s001.zip › Figure S09.tif]

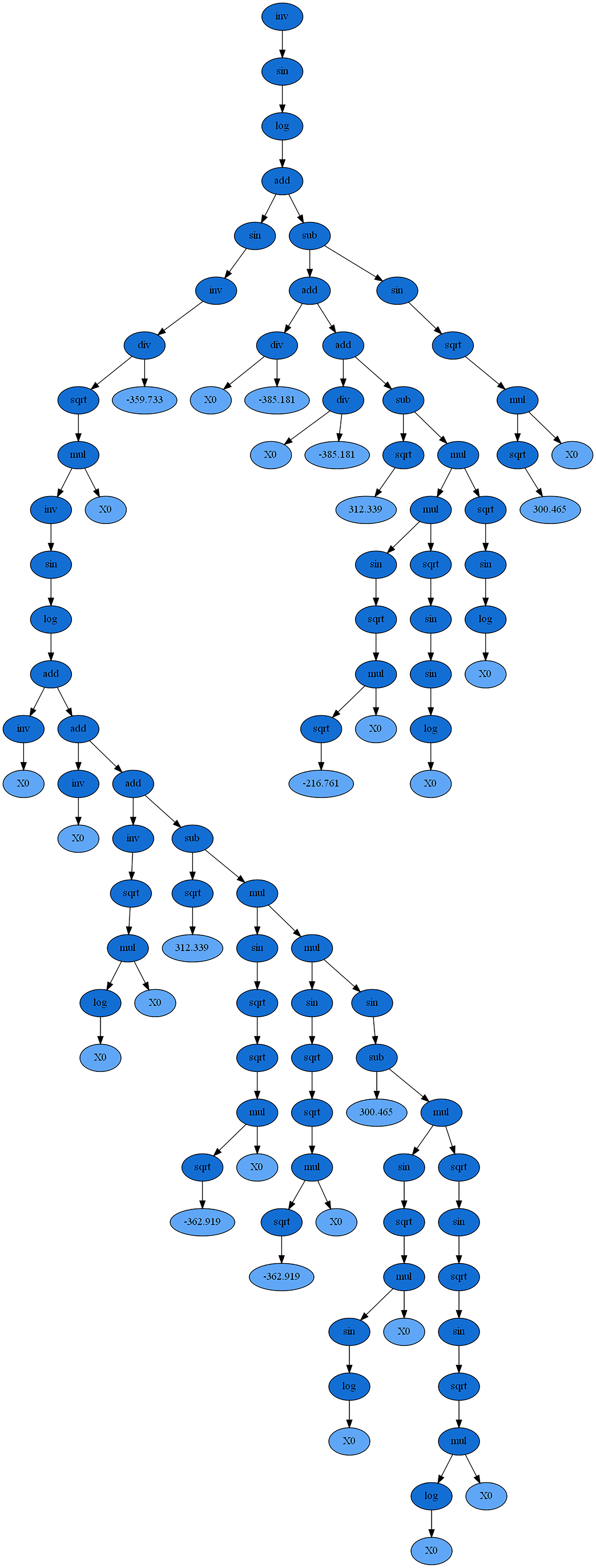

Supplement: Supplementary file 1 [file toxics-12-00354-s001.zip › Figure S10.tif]

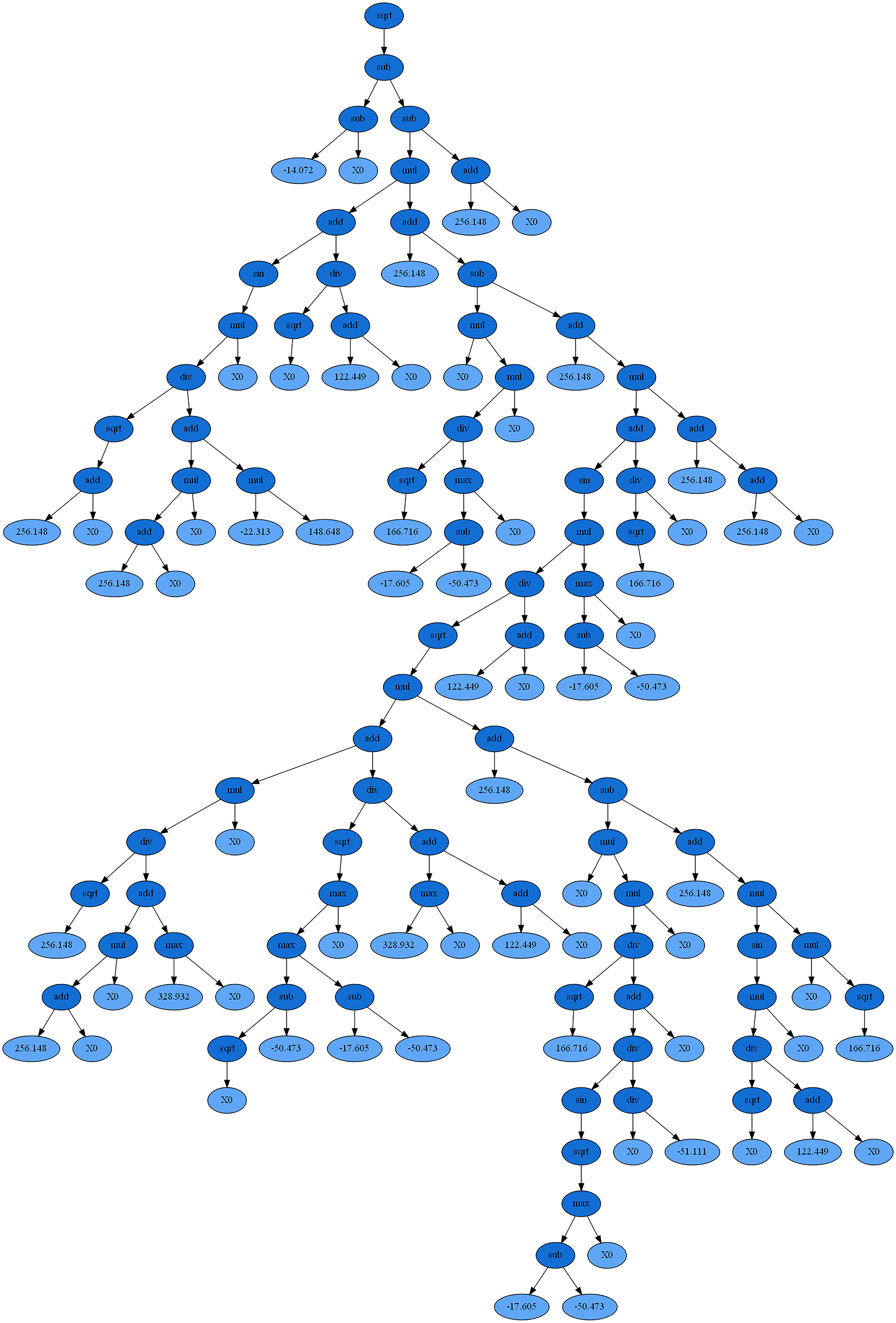

Supplement: Supplementary file 1 [file toxics-12-00354-s001.zip › Figure S11.tif]
